# Blood Pressure in Patients With Migraine Treated With Monoclonal Anti-CGRP (Receptor) Antibodies: A Prospective Follow-up Study

**DOI:** 10.1212/WNL.0000000000201008

**Published:** 2022-10-25

**Authors:** Simone de Vries Lentsch, Britt W.H. van der Arend, Antoinette Maassen VanDenBrink, Gisela M. Terwindt

**Affiliations:** From the Department of Neurology (S.d.V.L., B.W.H.v.d.A., G.M.T.), Leiden University Medical Centre; and Division of Vascular Medicine and Pharmacology (B.W.H.v.d.A., A.M.V.D.B.), Department of Internal Medicine, Erasmus University Medical Center, Rotterdam, the Netherlands.

## Abstract

**Background and Objectives:**

Anti–calcitonin gene-related peptide (CGRP) (receptor) antibodies are approved as preventive treatment for migraine. Recent concerns have been raised after a retrospective analysis of postmarketing case reports of elevated blood pressure (BP) associated with erenumab. In this prospective follow-up study, we aimed to assess the safety regarding BP in a real-world setting.

**Methods:**

All people with migraine who were treated with erenumab and fremanezumab at the Leiden Headache Center between January 2019 and January 2021 were included. BP measurements were collected from baseline (T0) until 12 months of follow-up, with a 3-month interval (T1–T4). Mixed linear models were fitted with time as a fixed effect and the patient as a random effect.

**Results:**

Both systolic and diastolic BP were increased at all time points T1–T4 compared with T0 (*p* < 0.001). The maximum estimated increase in the mean systolic BP was 5.2 mm Hg (95% CI 3.1–7.5). The maximum estimated increase in the mean diastolic BP was 3.5 mm Hg (95% CI 2.0–4.9). In the erenumab group (n = 109), both systolic and diastolic BP were increased at all time points compared with T0 (all *p* < 0.001). For fremanezumab (n = 87), systolic but not diastolic BP was increased compared with T0 at T1 (*p* = 0.006) and T2 (*p* = 0.004). Four patients (3.7%) with normal BP at T0 required antihypertensive treatment after erenumab was started.

**Discussion:**

The mean systolic and diastolic BP increased after anti-CGRP (receptor) antibodies were started. The majority of patients remained within the normal BP limits, but some patients required antihypertensive treatment. Physicians should be aware that people with migraine may be at risk of developing hypertension when treated with anti-CGRP (receptor) antibodies, and this should be added to (inter)national treatment guidelines.

**Classification of Evidence:**

This study provides Class III evidence that anti-CGRP (receptor) antibodies increase BP when used to treat patients with migraine.

Migraine is a primary headache disorder, characterized by recurrent episodes of moderate to severe headaches, accompanied by photo- and phonophobia and/or severe nausea and/or vomiting.^1^ The pathophysiology is not completely uncovered; however, calcitonin gene-related peptide (CGRP) has been identified to play a major role.^2^ Recently, new preventive migraine treatments targeting CGRP have become available: 3 monoclonal antibodies targeting the ligand CGRP (eptinezumab, fremanezumab, and galcanezumab) and 1 targeting the CGRP receptor (erenumab).

CGRP is known to be a potent vasodilator.^2^ Besides its role in migraine, CGRP is involved in blood pressure (BP) regulation.^3^ Therefore, the use of these monoclonal antibodies may potentially lead to hypertension. The randomized placebo-controlled clinical trials did not report an increased risk of hypertension or other cardiovascular disease.^4^ Nevertheless, recent concerns have been raised after a retrospective analysis of postmarketing case reports of elevated BP associated with erenumab.^5^ Sixty-one cases of elevated BP related to treatment with erenumab were reported to the Food and Drug Administration (FDA). In contrast, no such concern yet has been reported regarding a CGRP antibody. As migraine itself is associated with an increased risk for cardio- and cerebrovascular events,^6-8^ it is important that anti-CGRP treatment does not increase this risk even more. In this prospective follow-up study, we assessed whether treatment with the preventive drugs erenumab and fremanezumab changes systolic and diastolic BP in people with migraine during 1-year follow-up.

## Methods

### Participants

All people with migraine receiving treatment with erenumab or fremanezumab between January 2019 and January 2021 at the Leiden Headache Center, a national academic referral center in the Netherlands, were deemed eligible for participation. Patients were included if BP measurements were present at baseline and patients had a follow-up of at least 6 months. All patients were diagnosed with migraine by a neurology resident in consultation with a headache specialist or by a neurologist with headache expertise, according to the International Classification of Headache Disorders, third edition, criteria.^1^ None of the patients had medication overuse headache at treatment initiation. With restricted availability of erenumab and fremanezumab, we were able to include patients with ≥8 migraine days, who failed on ≥4 migraine preventives (meaning being ineffective, discontinued because of side effects, or being contraindicated), including at least a beta-blocker, candesartan, valproate, and topiramate. Erenumab could be prescribed to patients aged 18–65 years and fremanezumab to patients aged 18–70 years.

### Treatment

Patients treated with erenumab started with 70 mg, administered subcutaneously once every 4 weeks and optionally increased this to 140 mg after at least 3 months. This decision was based on a shared decision between patients' and physicians' impression of effectiveness. Fremanezumab was prescribed as 225 mg subcutaneous injection every 4 weeks. Patients were not treated with additional preventive treatment.

### Blood Pressure

BP measurements (mm Hg) were collected from the electronic patient records. Patients had a consultation at the Leiden Headache Center at start (T0) and approximately every 3 months until treatment was discontinued. As part of regular clinical care, BP was measured in sitting position during these consultations with an automatic BP device by the treating physician or a nurse. Data were collected from baseline (before starting treatment, T0) and every follow-up visit thereafter with a maximum follow-up of 12 months (T1–T4). Patients were excluded from analyses if baseline BP was measured while patients were still tapering off current migraine preventive treatment that may affect BP, such as beta-blockers or candesartan.

Patients who were not able to come to a physical visit (e.g., during coronovirus disease lockdown) were sometimes asked to measure their BP at home or at the general practitioner. However, only BP measurements obtained at the Leiden Headache Center were included in our analyses. Hypertension was not an exclusion criterium for starting treatment with erenumab or fremanezumab. Patients with elevated BP (according to the international BP guidelines^9,10^) at any time during treatment were referred to their general practitioner for additional measurements (e.g., 24-hour measurements) and received treatment if deemed necessary. If patients started treatment with antihypertensive drugs, the follow-up BP values thereafter were excluded from the analyses. If patients were already treated for hypertension before starting erenumab or fremanezumab, their measurements would only be included if there was no dose or drug change in their antihypertensive drugs.

### Control Group

As a control group, we included people with migraine of the Leiden Headache Center with similar distribution in sex, age, and migraine diagnosis. These patients did not use any migraine prophylactic treatment or other medication that would possibly influence their BP. BP was measured in these patients as part of regular care. We collected BP measurements from 2 different time points (time range 1–3 months).

### Statistics

The sample size was based on the available data. Baseline characteristics, including sex, age, headache diagnosis, baseline headache, and migraine days, were summarized using mean, SDs, frequencies, and proportions.

For both systolic and diastolic BP, a linear mixed model was fitted with time and treatment (erenumab or fremanezumab) as fixed effects and the patient as a random effect. For the primary outcome, these analyses were performed for the total study population. As a secondary analysis, the mixed models were repeated for erenumab and fremanezumab separately. The control group was analyzed in the same manner.

The number of patients with hypertension or a relevant increase in BP during the course of treatment was assessed in 3 ways: (1) patients who started treatment with antihypertensive drugs during treatment with erenumab or fremanezumab; (2) patients with a systolic BP ≥140 mm Hg and/or diastolic BP ≥90 mm Hg at any time during follow-up; and (3) patients with an increase in systolic BP ≥20 mm Hg and/or an increase in diastolic BP ≥10 mm Hg at any time during follow-up. The patients who started antihypertensive treatment for hypertension were described in detail, including but not limited to age, sex, body mass index (BMI), baseline BP, and maximum BP measured.

Missing values were not imputed. In all analyses, 2-sided *p* values <0.05 were considered significant. All statistical analyses were performed using IBM SPSS Statistics for Windows, version 25 (IBM Corp., Armonk, NY).

### Sensitivity Analysis

For a post hoc sensitivity analysis, missing BP measurements were handled using multiple imputation. Ten imputed data sets on systolic and diastolic BP were generated using automatic imputation. The linear mixed model as described above was repeated using the imputed data set.

### Standard Protocol Approvals, Registrations, and Patient Consents

This study was approved by the Medical Ethics Committee of the Leiden University Medical Center, and all patients provided written informed consent.

### Data Availability

The data that support the findings of this study are available from the corresponding author on reasonable request.

## Results

A total of 211 patients started treatment with the anti-CGRP (receptor) antibodies erenumab or fremanezumab. In 7 patients, baseline BP was missing, and in 8 patients, baseline BP was measured while they were still tapering off a beta-blocker or candesartan. Two patients were already treated for hypertension, but their medication did not change during the course of this study, and thus, these patients were included in the analyses. We included 109 patients who started treatment with erenumab and 87 patients who started treatment with fremanezumab.

Among the patients treated with erenumab, 93 (85%) were female, the average age was 42 years, and 55 (51%) patients had chronic migraine. Among the patients treated with fremanezumab, 74 (85%) were women, the average age was 45 years, and 48 (55%) patients had chronic migraine. Baseline characteristics for both study groups are presented in Table 1.

**Table 1:**
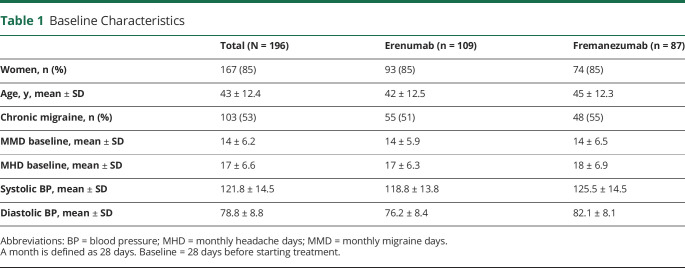
Baseline Characteristics

**Figure 1:**
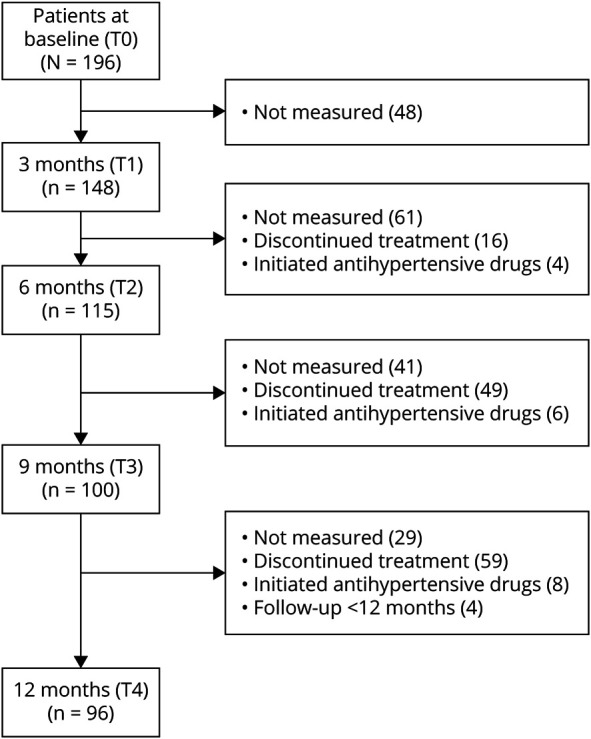
Flowchart Number of patients included in the analysis at every time point of the study and reasons for missing data. Left side: total number of patients available at the different time points. Right side: number of missing data with reasons for missing data The main reason for not measuring the blood pressure at follow-up was that these patients consulted our clinic through telemedicine (video consultation) due to lockdown measurements because of coronovirus disease 2019.

The number of patients of whom BP measurements were available at every time point is shown in the flowchart of Figure 1. Data could be missing because patients discontinued treatment, because the BP was not measured at the Leiden Headache Center, because patients' follow-up time was less than 12 months, or because BP treatment was initiated.

### BP in the Total Study Population

**Figure 2:**
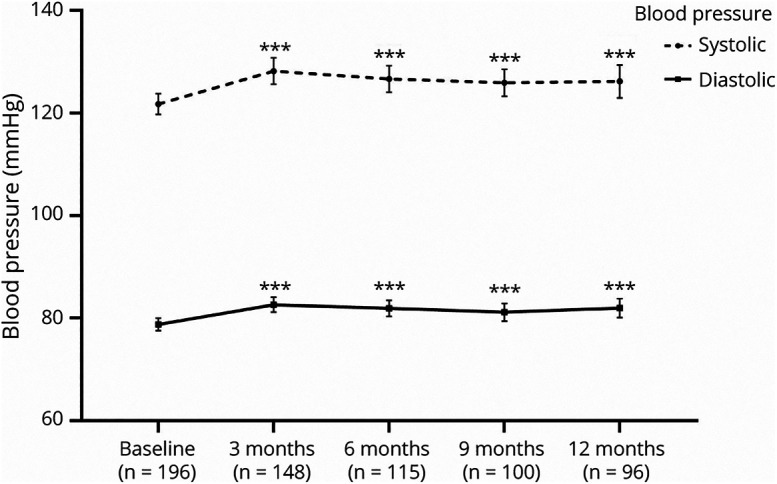
Blood Pressure Development During 12-Month Follow-up After Starting Erenumab or Fremanezumab Data presented in mean ± 95% CI. Asterisks present significant change compared with baseline: ***p < 0.001.

We observed an increase in systolic BP at all time points compared with baseline (Figure 2). At T1, systolic BP increased with 5.0 mm Hg (95% CI 3.1–6.9, *p* < 0.001). At T2, the estimated effect was 4.9 mm Hg (95% CI 2.9–7.0, *p* < 0.001). At T3, the estimated effect compared with baseline was 4.7 mm Hg (95% CI 2.5–6.9, *p* < 0.001). At T4, the systolic BP increased by 5.2 mm Hg (95% CI 3.1–7.5, *p* < 0.001). A larger estimated effect from erenumab than from fremanezumab was found for the increase in systolic BP (β ± SE = 4.3 ± 1.9, *p* = 0.03).

The diastolic BP increased as well at all time points compared with baseline (Figure 2). At T1, diastolic BP increased by 3.3 mm Hg (95% CI 2.1–4.5, *p* < 0.001). At T2, the estimated effect was 3.2 mm Hg (95% CI 1.8–4.5, *p* < 0.001). At T3, the estimated effect compared with baseline was 2.5 mm Hg (95% CI 1.0–3.9, *p* < 0.001). At T4, the diastolic BP increased by 3.5 mm Hg (95% CI 2.0–4.9, *p* < 0.001). A larger estimated effect from erenumab than from fremanezumab was found for the increase in diastolic BP (β ± SE = 2.4 ± 1.1, *p* = 0.03).

### BP in Patients Treated With Erenumab

**Figure 3:**
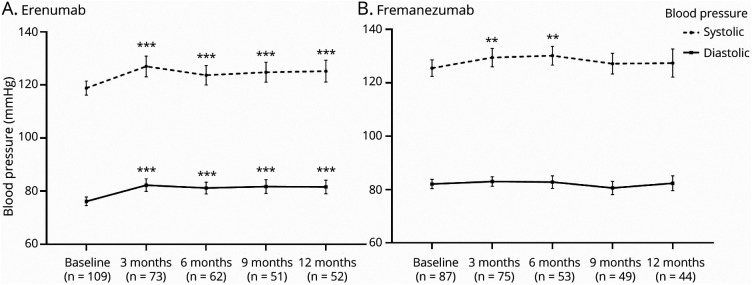
Blood Pressure Development During 12-Month Follow-up Separately for Erenumab and Fremanezumab Data presented in mean ± 95% CI for erenumab (A) and fremanezumab (B). Asterisks present significant change compared with baseline: **p < 0.01, and ***p < 0.001.

In the erenumab subgroup, we found an increase in systolic BP at all time points compared with baseline (Figure 3). At T1, the systolic BP increased by 5.8 mm Hg (95% CI 3.3–8.3, *p* < 0.001). At T2, the estimated effect was 5.0 mm Hg (95% CI 2.3–7.7, *p* < 0.001). At T3, the estimated effect was 7.6 mm Hg (95% CI 4.6–10.5, *p* < 0.001). At T4, the systolic BP increased by 9.1 mm Hg (95% CI 6.2–12.0, *p* < 0.001).

The diastolic BP increased as well at all time points compared with baseline (Figure 3). At T1, diastolic BP increased by 5.4 mm Hg (95% CI 3.7–7.1, *p* < 0.001). At T2, the estimated effect was 4.9 mm Hg (95% CI 3.1–6.8, *p* < 0.001). At T3, the estimated effect was 5.8 mm Hg (95% CI 3.9–7.8, *p* < 0.001). At T4, the diastolic BP increased by 6.3 mm Hg (95% CI 4.4–8.3, *p* < 0.001).

### BP in Patients Treated With Fremanezumab

For fremanezumab, we found an increase in systolic BP at T1 and T2 but not at T3 and T4 (Figure 3). At T1, systolic BP increased by 3.8 mm Hg (95% CI 1.1–6.6, *p* = 0.006). At T2, it increased by 4.6 mm Hg (95% CI 1.5–7.7, *p* = 0.004). At T3, the estimated effect compared with baseline was 1.6 mm Hg (95% CI −1.6 to 4.8, *p* = 0.31). At T4, the estimated effect was 0.9 mm Hg (95% CI −2.4 to 4.2, *p* = 0.59).

The diastolic BP in patients treated with fremanezumab did not increase (Figure 3). At T1, the mean difference was 0.8 mm Hg (95% CI −0.8 to 2.5, *p* = 0.33). At T2, the estimated effect was 0.8 mm Hg (95% CI −1.0 to 2.7, *p* = 0.39). At T3, the estimated effect compared with baseline was −1.4 mm Hg (95% CI −3.3 to 0.5, *p* = 0.16). At T4, the estimated effect was −0.02 mm Hg (95% CI −2.0 to 2.0, *p* = 0.99).

### Patients Starting Antihypertensive Drugs

In total, 9 patients started antihypertensive drugs during the course of treatment with erenumab (5/9) or fremanezumab (4/9). Five of these patients were referred to their general practitioner after the baseline BP measurement, but CGRP-blocking treatment was started before antihypertensive treatment was started. Four patients (3.7%) with normal BP at T0 required antihypertensive treatment after erenumab was started. Patient characteristics are provided in Table 2.

**Table 2:**
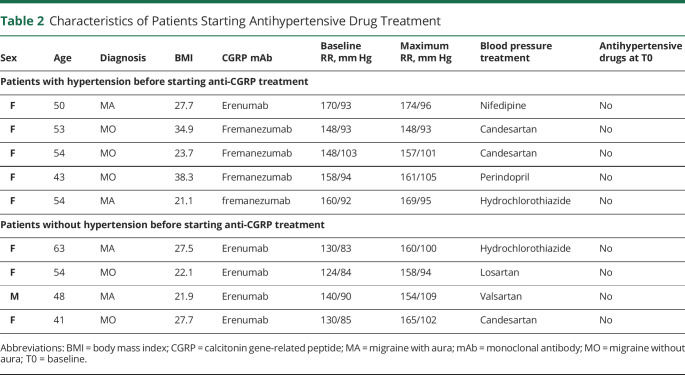
Characteristics of Patients Starting Antihypertensive Drug Treatment

### Patients With Elevated BP

Of all patients, 53 patients had a systolic BP ≥140 mm Hg and/or a diastolic BP ≥90 mm Hg at any time during course of treatment with erenumab (33/109) or fremanezumab (20/87), whereas at baseline, BP was <140/90 mm Hg. In total, 76 patients had a systolic BP rise of ≥20 mm Hg and/or a diastolic BP rise ≥10 mm Hg at any time during the course of treatment with erenumab (52/109, 47.7%) or fremanezumab (24/87, 27.6%). In about half of these patients (total 41 patients; erenumab 28 patients and fremanezumab 13 patients), this BP rise did not lead to a systolic BP ≥140 mm Hg and/or a diastolic BP ≥90 mm Hg.

### Control Group

A total of 109 people with migraine were included in the control group. The average systolic BP of the first measurement was 121.3 ± 14.9 mm Hg, and the average diastolic BP was 77.0 ± 9.9 mm Hg. The average follow-up systolic BP was 120.9 ± 14.8 mm Hg, and the average follow-up diastolic BP was 77.7 ± 8.7 mm Hg. There was no change over time in systolic (*p* = 0.70) or diastolic (*p* = 0.39) BP.

### Sensitivity Analysis

In the analysis with the imputed data set, the systolic BP increased at all time points compared with baseline. At T1, systolic BP increased with 5.3 mm Hg (95% CI 2.7–7.9, *p* < 0.001). At T2, the estimated effect was 4.3 mm Hg (95% CI 1.1–7.5, *p* = 0.009). At T3, the estimated effect compared with baseline was 4.3 mm Hg (95% CI 1.2–7.5, *p* = 0.004). At T4, the systolic BP increased by 3.7 mm Hg (95% CI 0.8–6.5, *p* = 0.01). A larger estimated effect from erenumab than from fremanezumab was found for the increase in systolic BP (β ± SE = 2.2 ± 0.8, *p* = 0.004).

Likewise, the diastolic BP increased at all time points compared with baseline. At T1, diastolic BP increased by 3.2 mm Hg (95% CI 1.6–4.7, *p* < 0.001). At T2, the estimated effect was 2.7 mm Hg (95% CI 0.7– 4.7, *p* = 0.01). At T3, the estimated effect compared with baseline was 2.1 mm Hg (95% CI 0.4–3.7, *p* = 0.01). At T4, the diastolic BP increased by 2.5 mm Hg (95% CI 0.6–4.4, *p* = 0.01). A larger estimated effect from erenumab than from fremanezumab was found for the increase in diastolic BP(β ± SE = 1.1 ± 0.5, *p* = 0.02).

### Classification of Evidence

This study provides Class III evidence that anti-CGRP (receptor) antibodies increase BP when used to treat patients with migraine.

## Discussion

We collected BP measurements in a prospective 1-year follow-up study of patients with migraine treated with erenumab and fremanezumab at the Leiden Headache Center. Already at the first follow-up visit, after 3 months of treatment, an increase in both mean systolic and mean diastolic BP was observed. Furthermore, the increase in the mean systolic and mean diastolic BP was a long-lasting effect, lasting the entire follow-up period of 12 months. For some patients (3.7% of the patients treated with erenumab), this required the start of antihypertensive treatment. Of all patients, 75 (38%) had a relevant increase in BP (i.e., ≥20 mm Hg systolic and/or ≥10 mm Hg diastolic) at any time during follow-up, whereas half of these patients remained within the normal BP limits.

In line with the clinical trials of erenumab and fremanezumab,^11,12^ we did not find a major risk of developing hypertension. Although, fortunately, the majority of patients did not require treatment for hypertension, we did observe a modest effect on the mean BP. It is important to realize that BP and cardiovascular events have a continuous relation.^13^ The cutoff values of hypertension are the levels of BP at which it was demonstrated that the benefits of treatment outweigh the risks of antihypertensive treatment.^9^ Previous studies indicated that CGRP does not seem to have a role in the physiologic regulation of normal BP,^3^ but that it provides a key compensatory mechanism against hypertension.^14^ Thus, although a potential risk of hypertension may arise when patients are treated with CGRP-blocking medication, it may be that blocking CGRP is only potentially problematic for patients already at risk of developing hypertension. After the first observed increase after 3 months of treatment, the mean systolic and diastolic BP remained stable. This is consistent with the fact that in the majority of cases reported to the FDA, the hypertension was detected in the first week(s) after the first erenumab injection.^5^ This suggests that whether a patient develops hypertension will be apparent soon after initiating treatment. At the same time, it seems that the rise in BP is a long-lasting effect of treatment and no adaptation process takes place within at least 12 months. This long-lasting effect, together with the results of our control group, also makes it less likely that the increase we found is due to natural fluctuation.

Our data suggest that there might be a different effect for erenumab than for fremanezumab. Our results seem to demonstrate a larger and more consistent effect on the BP in patients treated with erenumab than in patients treated with fremanezumab. We cannot for certain conclude whether there are indeed differences between these 2 drugs or what the reason for these differences would be. One reason could be that erenumab might have a larger inhibiting effect on the CGRP pathway than fremanezumab, although this seems unlikely given the similar clinical efficacy of both drugs. An alternative explanation arises from the fact that erenumab (an antibody against the CGRP receptor) and fremanezumab (an antibody against the peptide CGRP) affect the CGRP pathway in different ways, which could hypothetically lead to clinical differences due to differences in receptor internalization and/or action of other ligands on receptors from the CGRP family.^15,16^ A third possible explanation would be that there might have been a lack of statistical power in the analyses for the fremanezumab subgroup. In addition, we did not randomly assign patients to treatment with erenumab or fremanezumab. Although the baseline characteristics seem similar, it might be that there is a difference between the 2 patient populations. Finally, although for erenumab, previous data suggested an effect on BP, for fremanezumab, data are still limited. In a previous study, a single dose of fremanezumab did not cause any change in systolic or diastolic BP.^17^ However, the sample size in that study was extremely small, only 23 healthy women receiving different dosages of fremanezumab. A recent abstract presenting BP data of the clinical trials with fremanezumab described no changes in either systolic or diastolic BP.^18^ Unfortunately, no information was provided on the use and changes in dosage of BP medication. Further research is necessary to confirm the potential differences between erenumab and fremanezumab and to examine the possible explanations for such differences.

It may be that increasing the dosage from 70 to 140 mg would increase the BP further in patients. However, although the majority of patients did increase the dosage to 140 mg at some point during follow-up, we did not see a significant increase in month 6, 9, or 12 compared with month 3. However, for these analyses, our study might be underpowered. An additional limitation of this study is the risk of a white coat effect when measuring BP in the doctor's office.^19^ This could have led to an overestimation of the number of patients with a BP ≥140/90 mm Hg. However, by including exclusively values measured at the Leiden Headache Center, we intended to reduce the variability and to remain the reliability of the measurements. All BP measurements were performed by a health care professional with the same type of BP device. If there were a white coat effect, it would account also for the baseline measurement and therefore cannot be an explanation for the increase in BP. In addition, antihypertensive medication was not prescribed based on the BP measured at the headache center. The general practitioner made this decision based on additional measurements and BP guidelines.^10,20^ A third potential limitation of this study comes from excluding BP measurements after patients started treatment for hypertension. However, this probably caused an underestimation of the rise in BP and thus is unlikely to affect our conclusions. Finally, we realize that the missing data could have influenced the results. Therefore, we performed a sensitivity analysis, after multiple imputation, which did not demonstrate different results.

In our patient population, only 3.7% of patients treated with erenumab had new-onset hypertension requiring treatment, which is not sufficient to identify patient-specific risk factors. Currently known risk factors for hypertension in the general population include modifiable risk factors such as higher BMI and smoking and unmodifiable risk factors such as higher age, family history of hypertension, and coexisting diseases such as diabetes and kidney disease. It is uncertain whether the same risk factors can be applied to this specific patient group. Of interest, the age range of the postmarketing cases reported at the FDA was 24–88, suggesting that not only older patients are at risk.^5^ In addition, a seemingly obvious risk factor would be tapering off preventive migraine treatment with antihypertensive properties, such as candesartan and beta-blockers, before starting treatment with anti-CGRP (receptor) antibodies. However, as mentioned above, this was an exclusion criterion on our study, and therefore, this was not the case in any of the patients in our study who developed hypertension requiring treatment. Furthermore, nonsteroidal anti-inflammatory drugs are known to be able to increase BP. However, as acute medication use diminished during follow-up,^21^ this is not likely a cause for the increase in BP. More real-life data might help to identify patient-specific risk factors for developing hypertension regarding treatment with CGRP (receptor)-targeting antibodies, which will be important to include in clinical treatment guidelines. We believe that it is of utmost importance to monitor BP in real-world settings in patients treated with CGRP-targeting drugs. Hypertension in people with migraine is suggested to be associated with conversion from episodic to chronic migraine.^22^ Moreover, people with migraine, especially women and those people with migraine with aura, already have a vascular vulnerability^23^ with an increased risk of white matter lesions,^24,25^ stroke,^7,8^ and coronary heart disease^6^ with underlying shared genetic mechanisms.^26,27^ Hypertension is known to be the most important modifiable risk factor for cardiovascular disease.^28^ Therefore, timely detection of elevated BP is essential for adequate intervention.

The mean systolic and diastolic BP slightly increased after starting treatment with erenumab or fremanezumab. Fortunately, the majority of patients did not require treatment with antihypertensive drugs. As CGRP appears to provide a protective mechanism in hypertension, blocking CGRP could be specifically problematic for patients already at risk of developing hypertension. As migraine itself is associated with an increased risk for cardio- and cerebrovascular events, it is important to monitor the BP after starting treatment with CGRP targeting treatment to prevent increasing this risk. Physicians should be aware of the possibility that people with migraine develop hypertension when treating them with anti-CGRP (receptor) antibodies. Caution for raised BP regarding anti-CGRP (receptor) antibodies should be added to clinical treatment guidelines for migraine.

## Glossary

BMI: body mass index
BP: blood pressure
CGRP: calcitonin gene-related peptide
FDA: Food and Drug Administration
